# Why were there 231 707 more deaths than expected in England between 2010 and 2018? An ecological analysis of mortality records

**DOI:** 10.1093/pubmed/fdab023

**Published:** 2021-03-26

**Authors:** Frances Darlington-Pollock, Mark A Green, Ludi Simpson

**Affiliations:** Department of Geography and Planning, University of Liverpool, L69 7ZT Liverpool, UK; Department of Geography and Planning, University of Liverpool, L69 7ZT Liverpool, UK; Cathie Marsh Institute for Social Research, University of Manchester, M13 9BL, Manchester, UK

## Abstract

**Background:**

Policy responses to the Global Financial Crisis emphasized wide-ranging fiscal austerity measures, many of which have been found to negatively impact health outcomes. This paper investigates change in patterns of mortality at local authority level in England (2010–11 to 2017–18) and the relation with fiscal austerity measures.

**Methods:**

Data from official local authority administrative records are used to quantify the gap between observed deaths and what was anticipated in the 2010-based subnational population projections. Regression analyses are used to explore the relation between excess deaths, austerity and wider process of population change at local authority level.

**Results:**

We estimate 231 707 total excess deaths, the majority of which occurred since 2014–15 (89%) across the majority of local authorities (91%). Austerity is positively associated with excess deaths. For working age adults, there is a clear gradient to the impact of austerity, whereas for older adults, the impact is more uniform.

**Conclusions:**

Fiscal austerity policies contributed to an excess of deaths for older people and widened social inequalities for younger populations. These results call for an end to all austerity measures and require further research into areas with the highest total excess deaths as a priority following the COVID-19 pandemic.

## Introduction

The global financial recession of 2008 heralded a rise of fiscal austerity to reduce national deficits through varying combinations of expenditure cuts. In the UK, post-2010 fiscal policy constrained health and social care budgets, while decimating local government spending.^1^^,^^2^ Deprived people and deprived places suffered most, bearing the brunt of cuts to welfare provision and local authority service expenditure.^1^^,^^3–5^

The erosion of the basic principles of a welfare state, specifically the public responsibility to provide for those in need, has served as the backdrop for debates on recent trends in stalling life expectancy emerging since 2014.^6^ This was thrown into sharper relief as 2015 saw one of the largest annual increases in mortality since World War II,^7^ particularly amongst older age groups (70+ and 80+).^8^ This spike in older ages has been attributed to austerity measures leading to changes in income support for poor pensioners and spending reductions on social care.^9^ While many echo concerns over the impacts of such austerity measures,^10–12^ alternative explanations include statistical artefact, influenza, cold weather and even new infectious agents.^13–16^

We estimate the scale of change in mortality trends culminating in declining life expectancy during this period of fiscal austerity. The aim of our study is to investigate the gap between observed patterns of mortality and what was anticipated in the 2010-based subnational population projections (SNPPs) at local authority level. Population projections are core resources informing planning and decision-making across the breadth of public policy.^17^^,^^18^ Any significant changes emerging in the demographic components of population change must then be evaluated, particularly if such changes coincide with the type policy shift as observed in 2010. Local authorities are a key conduit by which services and resources are channelled into local populations, each of which vary significantly in their age–sex structure and level of socioeconomic deprivation.^19^^,^^20^ Understanding the extent of mortality change and which areas were affected most is key for evaluating the experiences of population groups. We are not aware of any study that has estimated or explored these issues.

## Methods

Subnational population projections are released every 2–3 years by the Office for National Statistics (ONS). Using the cohort component method, these model change in the age–sex structure of the population over time as well as detailed components of demographic change: births, deaths and migration.^21^ Using an initial starting population (e.g. 2010 mid-year population estimates), the population is aged forward annually while accounting for new births, deaths, in- and out-migrations.

First, we calculate excess deaths. Excess deaths, as defined in this paper, quantifies the gap between the anticipated vitality of the population according to the 2010-based SNPPs and subsequent reality as recorded in the mid-year population estimates. For the latter, we use the mid-2018 release, which includes a time series of the components of population change since mid-2001.^21^ For the former, the ONS release expected counts of deaths according to the SNPPs by local authority, sex and single year of age, rather than the projected mortality rate.^22^ To avoid confounding errors in projected mortality with errors in projected migrations (which can have a significant impact on population size and therefore number of deaths), the projected counts of expected deaths are adjusted, multiplying the expected deaths by the mid-year population estimates over the projected population counts. Hereafter, reference to ‘projected deaths’ relates to our adjusted figure, rather than the raw numbers of deaths projected by the ONS. Excess deaths during the 12 months up to mid-year $y$ are calculated as:

$$\begin{align*} &{ExcessDeaths}_y={ObservedDeaths}_{a,s,i,y}\\&-\left[{ProjectedDeaths}_{a,s,i,y}\times \frac{MYE_{a-1,s,i,y-1}}{P{rojectedPopulation}_{a-1,s,i,y-1}}\right] \end{align*}$$

where, $y$ = year, $a$ = age in completed years at mid-year, $s$ = sex and $i$ = location.

The excess deaths are summed over age, by local authority and sex. The results are expressed as a percentage where the number of excess deaths is divided by the number of projected deaths.

Second, we evaluate the association between total excess deaths (as a percentage of projected deaths) at the local authority level and austerity and wider processes of population change. We hypothesize that areas with greater levels of welfare cuts due to post-2010 fiscal austerity measures will have greater excess deaths. We measured fiscal austerity using Beatty and Fothergill’s^4^ estimates of the average impact of welfare reforms (pre- and post-2015) on household finances. This measure provides a clear pathway for how fiscal austerity may impact mortality patterns. The long established importance of income and income inequality as a social determinant of health^23^ would suggest that reduced economic resources may directly and indirectly affect health and health behaviours influencing mortality patterns. Although our measure projects financial impacts forward to 2020/21, it is indicative of the severity and differentiation of austerity measures between Local Authorities.

We also hypothesize higher excess deaths in the most deprived areas pre-austerity given the heightened vulnerability of populations living in more deprived areas. Living in a more deprived area is associated with poorer chances of good health, increased risk of negative health-related behaviours and increased mortality.^24–28^ The pathways linking poorer health outcomes to deprivation vary and may include differences in the availability of local resources or differences in exposure to local hazards or assets. We captured deprivation using the 2010 Index of Multiple Deprivation (IMD).^29^ IMD is the preferred indicator used by government for measuring deprivation and is used in resource allocation decisions. IMD is a multidimensional index covering seven domains including employment, income and education. We used the average rank of overall score across the local areas within a local authority as our explanatory variable. The more deprived an area is, the lower the average rank.

Finally, we hypothesize that the relationship between area deprivation, fiscal austerity and excess deaths will vary according to the local age–sex structure of the population and patterns of population change. The mortality profile of a more youthful population will be significantly different from that of on older population, but this will be complicated by differences in migration patterns between areas.^30^ The age profile of migrants into and out of an area between 2010 and 2018 may therefore influence differences in area-level mortality (e.g. high in-migration of older populations may increase mortality rates and excess deaths). Capturing these complex demographic processes is difficult and we have focused on four main elements of population change. Total proportion of the population aged 65 and over in 2010 was included due to established elevated risk of mortality in older ages. Change in net migration for ages 16–29 and ages 65 and over were included to account for potential changes to population composition that may have increased (i.e. in-migration of older groups) or decreased (i.e. in-migration of young populations) mortality rates. Finally, change in the dependency ratio for children relative to working age adults (neontic) and older people relative to working age adults (gerontic) were included to capture changes in age structure of areas. Dependency ratios are calculated as the ratio of the dependent group (whether children or older people) to the working age population (ages 15–64).

Statistical analyses included descriptive statistics and correlation coefficients to summarize overall patterns. We visualized raw estimates as well as calculated the smoothed conditional mean using LOESS regression to generalize patterns. OLS regression models were used to assess associations between our explanatory variables and excess deaths. Models were stratified by sex. All analyses were undertaken in R.

Ethical approval was not required for the secondary analysis of open datasets.

## Results

Table 1 summarizes average excess deaths as a percentage of the projected deaths since 2010–11, and the annual count of excess deaths for the total population. 231 707 more people died in the period between 2010–11 and 2017–18 than anticipated (Table 1). In total, 89% of these excess deaths have occurred since 2014–15, including 70 140 in 2017–18 (30% of the total excess deaths in the 8 years).

**Fig. 1:**
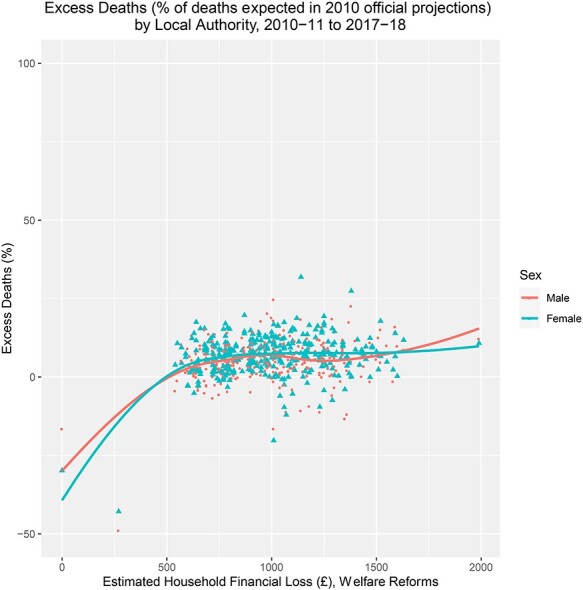
Excess deaths by local authority and sex, and average estimated financial household loss (£) through welfare reforms, 2010–11 and 2017–18. Note: Excess deaths is the total excess deaths between 2010–11 and 2017–18 as a % of projected deaths for each local authority, stratified by sex.

**Table 1:** Excess deaths, 2010–11 to 2017–18

	*2010–11*	*2011–12*	*2012–13*	*2013–14*	*2014–15*	*2015–16*	*2016–17*	*2017–18*
Average excess deaths								
Total	0%	0%	5%	0%	10%	8%	11%	15%
Male	0%	0%	4%	0%	9%	7%	10%	14%
Female	0%	1%	6%	-1%	12%	8%	12%	16%
Total excess deaths (231 708)								
	1116	1877	22 858	535	47 615	36 563	51 003	70 140

**Table 2:** Explaining excess deaths at the local authority level by sex, 2010–11 to 2017–18

	*B*	*L CI*	*U CI*	*Sig.*
Intercept				
	−9.86	−14.08	−5.63	^*^^*^^*^
	−5.09	−9.83	−0.79	^*^
Welfare reform: average estimated household loss				
(m)	0.01	0.00	0.01	^*^^*^^*^
(f)	0.01	0.00	0.01	^*^^*^^*^
Proportion aged 65+ (2010)				
(m)	0.47	0.33	0.60	^*^^*^^*^
(f)	0.28	0.17	0.39	^*^^*^^*^
Net change migration (65+)				
(m)	−342.37	−496.83	−187.91	^*^^*^^*^
(f)	−194.15	−340.08	−48.21	^*^
Change in neontic dependency ratio				
(m)	−0.09	−0.59	0.41	
(f)	−0.62	−1.13	−0.12	^*^
	*R* ^2^		Adjusted *R*^2^	
(m)	17.1		16.06	
(f)	13.36		12.28	

Significance codes: 0 ‘^*^^*^^*^’ 0.001 ‘^*^^*^^*^’ 0.01 ‘^*^^*^’ 0.05 ‘.’ 0.1 ‘ ’ 1. Note: *n* = 325 Local Authorities, Isles of Scilly excluded due to missing data.

The average excess deaths across local authorities is 6.04%. In total, 91% of local authorities had >0 excess deaths (91% and 87% for females and males, respectively). We found no association between excess deaths and deprivation (${r}_{\mathrm{s}}$ = 0.02), but a clearer pattern emerged in respect of the average household financial impact of welfare reforms, particularly when stratifying by age. Figure 1 plots total excess deaths (as a percentage of projected deaths) by local authority and sex against the financial impact of welfare reforms. There is a weak positive association between levels of excess deaths and the impact of the welfare reforms (${r}_{\mathrm{s}}$ = 0.21). This suggests that increases in the average estimated financial household loss at local authority level were associated with small increases in the level of excess deaths.

**Fig. 2:**
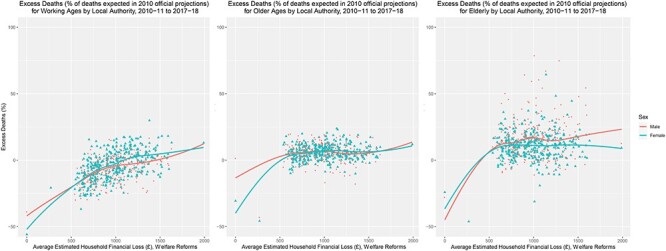
Excess deaths by local authority, sex and age groups, and average estimated financial household loss (£) through welfare reforms, 2010–11 and 2017–18. Note: Excess deaths is the total excess deaths between 2010–11 and 2017–18 as a % of projected deaths for each local authority, stratified by sex and age groups.

Figure 2 stratifies by age, differentiating between working ages (15–64), older ages (65–84) and elderly (85+). Since excess deaths are expressed as a percentage, its greater size at older ages indicates that those already vulnerable to mortality experience a greater increase of risk than others. For working age adults, there is a clear gradient (${r}_{\mathrm{s}}$ = 0.52) with higher excess deaths in areas where the financial impact of the welfare reforms has been greater. The relationship for the older age groups was weaker (${r}_{\mathrm{s}}$ = 0.14 for both groups). More than 85% of local authorities saw >0 excess deaths for ages 65–84, rising to ~90% for ages 85+. For working age adults, 38% of local authorities saw >0 excess deaths for women compared with 28% for men.

**Fig. 3:**
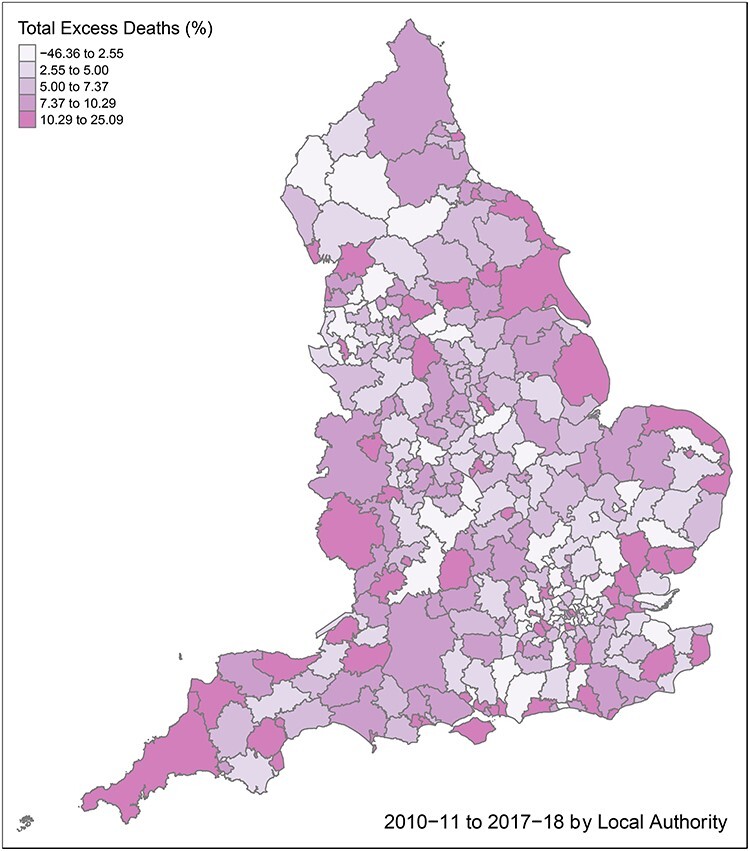
Total excess deaths as a percentage of projected deaths, between 2010–11 and 2017–18, by local authority.

Figure 3 maps total excess deaths (as a percentage of projected deaths) between 2010–11 and 2017–18, illustrating the consistency to excess deaths across England. Despite few distinct geographical patterns, coastal areas have a tendency towards higher excess deaths. Excess deaths also increase radiating out from Greater London, notwithstanding some London boroughs with higher excess levels.

Next, we examined the area-level determinants of the spatial distribution of total excess deaths through linear regression. Table 2 presents the final analytical model (model building process outlined in the Supplementary Material A1). Deprivation is not included in the final model due to the strength of the interrelationships with the financial impact of the welfare reforms.^1^^,^^4^ This was indicated by a high Variance Inflation Factor > 5 for financial impact of the welfare reforms (both men and women), and >6 for deprivation (both men and women) in model 5 (Supplementary Material A1). Excluding deprivation from the final analytical model minimized multicollinearity issues.

For men and women, as the financial impact of welfare reforms increases, so does excess deaths (p < 0.001). Adjusting for wider indicators of population change (Models 1–5) was found to elevate rather than negate the influence of welfare reforms. As the proportion of the population aged 65 and over increases, so too does excess deaths (*P* < 0.001). However, as net migration of older adults increases, excess deaths decreases (*P* < 0.001 for men, *P* < 0.05 for women). Preliminary modelling suggested a similar relationship between net migration of younger people and excess deaths for men (model 3), this relationship disappeared when controlling for changes in dependency ratios (model 4). For women, increases in the neontic dependency ratio are associated with decreases in excess deaths (*P* < 0.05). In the final model, change in the proportion of the population aged 65 and over exerts the greatest influence on differences in excess deaths at local authority level for both men and women, although the effect is greater for men. For example, predicted excess deaths at the 5th and 95th percentile for the population aged 65 and over varies from 1.73% to 9.83% for men and 4% to 9.7% for women. Change in the financial impact of welfare reform saw similar variability for men and women across local authorities (3.19% to 8.7% for males, 4.56% to 9.69% for women). There was far less variability in the influence of either change in net migration of older adults or the dependency ratio for women, although the influence of net migration change on differences in excess deaths at local authority level for men was more marked.

## Discussion

### Main finding of this study

Since 2010–11, we estimate that 231 707 more people died compared with what was anticipated in the 2010-based projections. The majority of excess deaths have occurred since 2014–15 (89%) and have occurred in most local authorities (91%). We find evidence that the impact of austerity is positively associated with total excess deaths, with more deaths in areas where the financial impact on households was, on average, greater. While deaths were also greater in areas with older populations, changes in the age structure of the areas over the time period were important.

Although deprivation matters for some (e.g. working age adults), estimates of average household financial loss attributed to changes in welfare provision are significant in shaping differences in excess deaths. Average household financial loss may reflect a more direct pathway as to how austerity policies matter for changing mortality profiles than wider socio-economic deprivation reflecting resources and assets available to individuals in local areas.^31^ Our results suggest the continuation of or introduction of new fiscal austerity reforms will simply reinforce inequalities and cannot be justifiable on welfare grounds.

An older population at baseline, as well as net migration patterns for older people, was also important in explaining total excess deaths, although variation in the proportion of the older population at baseline exerted greater influence on excess deaths than differences in net migration by local authority. This raises questions as to the differential vulnerability of older populations to significant changes in welfare provision. Similar findings have been highlighted elsewhere.^9^^,^^12^ While our findings evidence the impact on older populations, we demonstrate a clear social gradient in how patterns of total excess deaths have affected working age populations (Fig. 2).

### What is already known on this topic

Despite more than a century of sustained progress, improvements in life expectancy have stalled in the UK, coincident with a marked spike in mortality for older people in 2015. Although part of this spike is attributable to the mismatch between the circulating influenza strain in 2014–15 and the available vaccine, this was otherwise a mild flu season.^32^ Similarly, while many European countries have also experienced slowdowns to annual life expectancy improvements,^33^ these were not as severe as that experienced in England. This suggests the importance of the context of England (and the UK). Such dramatic changes are typically seen in times of crisis, whether war, famine or pandemic. Yet, for 2017–18, excess mortality equated to 70 140 deaths, exceeding the current estimates of lives lost to COVID-10 (as of 9 July 2020). Our results follow an emerging body of evidence demonstrating the negative impacts of constrained health and social care budgets, welfare reforms and the precarity induced by the economic recession and recovery.^10^^,^^34–36^ While austerity policies pursued since 2010 have been identified as one contribution to changing mortality patterns, the scale of change in mortality compared with what was anticipated in the 2010-based SNPPs has not previously been established.

### What this study adds

Constant update and evaluate of population forecasts is a core tenet in public policy decision making, especially in light of substantial changes to recent mortality trends.^37^ Our findings help unpack important mechanisms for evaluating the context of policy development and explore whether and how gaps between expectations and reality arise. The significance of our estimated 231 707 excess deaths is clearly underlined in that 91% of local authorities saw an increase in observed deaths. Social inequalities have widened between 2010–11 and 2017–18 for younger people, even if they constitute a smaller proportion of total number of excess deaths. This follows years of narrowing inequalities,^38^ suggesting the gains of considerable policy investment have been lost. Although the weaker association between average estimated household financial loss at local authority level and degree of excess deaths for people aged 65 and over may reflect some protection offered by the triple lock policy on pensions implemented in 2011—a guarantee for an annual increase in the basic state pension—there is still evidence of a harmful relationship between austerity and mortality for older ages as previous research has suggested.^9^

If there is a causal relationship between the post-2010 austerity measures and changing patterns of mortality, it is clear that the impact stretches beyond vulnerable places or vulnerable older people alone (although both appear correlated to the scale of impacts). The relatively weak relationship between deprivation and total excess deaths is indicative of a mechanism operating at the population level, rather than through more expected routes via social and spatial determinants of health.

### Limitations of this study

Our study utilizes longitudinal data on official local authority administrative records to calculate excess deaths. However, these data are ecological and do not relate to individuals. While our regression analyses help tease out the correlating factors to excess deaths, as a cross-sectional study it cannot tease out casual relationships. To identify and inform future policy approaches, we must move beyond our descriptive approach to formally test hypotheses, as well as developing more holistic measures capturing the impacts of austerity on people and places. While we look at total excess deaths we do not investigate (due to data availability) how excess deaths varied by cause of death. This is important to unpack the pathways and drivers of inequalities. Finally, the validity of population projections decrease over time and some degree of divergence is to be expected.^39^ Typically, life expectancy is consistently under projected in official projections,^40^ suggesting that our estimates may actually underestimate the true divergence in trends.

## Conclusions

We estimate a total of 231 707 excess deaths have occurred in England between 2010–11 and 2017–18. The scale of these excess deaths is almost equivalent to having a global pandemic in COVID-19 annually. The commencement of our study period coincides with the end of policies explicitly targeting health inequality^2^^,^^38^ and a significant shift in fiscal policies considered necessary to reduce the national deficit.^1^ This period has been characterized by a marked lack of progress relative to Marmot’s landmark review of health inequalities in and poorer performance on measures of mortality relative to our socioeconomic peers.^41^ As England and others seek to recover from the devastation of COVID-19 and grapple with an impending recession,^42^ these results must inform decisions as to the nature of any fiscal recovery package. Reversing these worrying mortality trends through ending austerity policies and investing in areas with highest total excess deaths should become the government’s priority following the COVID-19 pandemic.

## Supplementary Material

Appendix_fdab023

## Notes

**Frances Darlington-Pollock**, Lecturer

**Mark A. Green**, Senior Lecturer

**Ludi Simpson**, Honorary Professor

## References

[1] Gray M , Barford A. The depths of the cuts: the uneven geography of local government austerity. Cambridge J Reg Econ Soc 2018;11:541–63.

[2] Barr B , Bambra C, Whitehead M et al. The impact of NHS resource allocation policy on health inequalities in England 2001-11: longitudinal ecological study. BMJ 2014;348:1–10. 10.1136/bmj.g3231 PMC4035504 24865459

[3] Hastings A , Bailey N, Bramley G et al. The Cost of the Cuts: The Impact on Local Government and Poorer Comunities. York: Joseph Rowntree Found, 2015, 5–127.

[4] Beatty C , Fothergill S. The Uneven Impact of Welfare Reform: The Financial Losses to Places and People, 2016, 88. 10.7190/cresr.2017.5563239352.

[5] Centre for Cities . Cities Outlook 2019. 2019. https://www.centreforcities.org/reader/cities-outlook-2019/ (1 September 2020, date last accessed).

[6] Hiam L , Dorling D, McKee M. The cuts and poor health: when and how can we say that one thing causes another? J R Soc Med 2018;111:199–202. 29877771 10.1177/0141076818779237 PMC6022891

[7] Hiam L , Dorling D, Harrison D et al. Why has mortality in England and Wales been increasing? An iterative demographic analysis. J R Soc Med 2017;110:153–62. 28208027 10.1177/0141076817693599 PMC5407517

[8] Green M , Dorling D, Minton J. The geography of a rapid rise in elderly mortality in England and Wales, 2014-15. Health Place 2017;44:77–85. 28199896 10.1016/j.healthplace.2017.02.002

[9] Loopstra R , McKee M, Katikireddi SV et al. Austerity and old-age mortality in England: a longitudinal cross-local area analysis, 2007–2013. J R Soc Med 2016;109:109–16. 26980412 10.1177/0141076816632215 PMC4794969

[10] Hiam L , Dorling D, Harrison D et al. What caused the spike in mortality in England and Wales in January 2015? J R Soc Med 2017;110:131–7. 28208024 10.1177/0141076817693600 PMC5407518

[11] Watkins J , Wulaningsih W, Da Zhou C et al. Effects of health and social care spending constraints on mortality in England: a time trend analysis. BMJ Open 2017;7:e017722. doi: 10.1136/bmjopen-2017-017722. PMC5719267 29141897

[12] Green MA , Dorling D, Minton J et al. Could the rise in mortality rates since 2015 be explained by changes in the number of delayed discharges of NHS patients? J Epidemiol Community Health 2017;71:1068–71. 28970194 10.1136/jech-2017-209403 PMC5847097

[13] Vestergaard LS et al. Excess all-cause and influenza-attributable mortality in Europe, December 2016 to February 2017. Euro Surveill 2017;22:1–6. 10.2807/1560-7917.ES.2017.22.14.30506 PMC5388126 28424146

[14] Milne E . Mortality in England – erroneous attribution of excess winter deaths to underlying trend. J R Soc Med 2017;110:264–6. 28718383 10.1177/0141076817703865 PMC5524254

[15] Newton J , Baker A, Fitzpatrick J et al. What’s Happening with Mortality Rates in England? Public Health Matters. 2017. https://publichealthmatters.blog.gov.uk/2017/07/20/whats-happening-with-mortality-rates-in-england/ (1 September 2020, date last accessed).

[16] Jones RP . Essays on rising mortality in England and Wales – a MEDLINE search is not infallible. J R Soc Med 2017;110:224. 28627993 10.1177/0141076817703864 PMC5499565

[17] Berrington A , Simpson L. Housing composition and housing need. In: Champion T, Falkingham J (eds). Population Change in the United Kingdom. London: Brown and Littlefield, 2016, 104–24.

[18] Wilson T , Rees P. Recent developments in population projections methodology: a review. Popul Space Place 2005;11:337–60.

[19] MHCLG . The English Indices of Deprivation Things You Need to Know. 2019. https://www.gov.uk/government/statistics/english-indices-of-deprivation-2019 (1 September 2020, date last accessed).

[20] Evandrou M , Falkingham J, León MG, et al. Local Government and the Demography of Ageing: Need to Know Review Number Five. 2015.

[21] Office for National Statistics . Estimates of the population for the UK, England and Wales, Scotland and Northern Ireland, 2020. https://www.ons.gov.uk/peoplepopulationandcommunity/populationandmigration/populationestimates/datasets/populationestimatesforukenglandandwalesscotlandandnorthernireland (8 June 2020, date last accessed).

[22] Office for National Statistics . 2008-based and 2010-based subnational population projections: deaths by age, sex and local authority. 2019. https://www.ons.gov.uk/peoplepopulationandcommunity/populationandmigration/populationprojections/adhocs/0097582008basedand2010basedsubnationalpopulationprojectionsdeathsbyagesexandlocalauthority (1 August 2020, date last accessed).

[23] Wilkinson RG . Income distribution and life expectancy. BMJ 1992;304:165–8. 1637372 10.1136/bmj.304.6820.165 PMC1881178

[24] Foster HME , Celis-Morales CA, Nicholl BI et al. The effect of socioeconomic deprivation on the association between an extended measurement of unhealthy lifestyle factors and health outcomes: a prospective analysis of the UK Biobank cohort. Lancet Public Health 2018;3:e576–85. 30467019 10.1016/S2468-2667(18)30200-7

[25] Stafford M , Marmot M. Neighbourhood deprivation and health: does it affect us all equally? Int J Epidemiol 2003;32:357–66. 12777420 10.1093/ije/dyg084

[26] Norman P , Boyle P, Rees P. Selective migration, health and deprivation: a longitudinal analysis. Soc Sci Med 2005;60:2755–71. 15820585 10.1016/j.socscimed.2004.11.008

[27] Morris R , Carstairs V. Which deprivation: a comparison of selected deprivation indexes. J Public Health Med 1991;13:318–26. 1764290

[28] McCartney G , Popham F, Katikireddi SV et al. How do trends in mortality inequalities by deprivation and education in Scotland and England & Wales compare? A repeat cross-sectional study. BMJ Open 2017;7:1–6. 10.1136/bmjopen-2017-017590 PMC5642664 28733304

[29] MHCLG . Index of Multiple Deprivation 2010. 2011. https://www.gov.uk/government/statistics/english-indices-of-deprivation-2010 (1 August 2020, date last accessed).

[30] Darlington-Pollock F , Norman P, Ballas D. Using Census Microdata to Explore the Inter-relationships Between Ethnicity, Health, Socioeconomic Factors and Internal Migration. In: Stillwell J (ed). Routledge Handbook of Census Resources, Methods and Applications. Unlocking the UK Census. Abindgon: Routledge, 2017.

[31] Wickham S , Bentley L, Rose T et al. Effects on mental health of a UK welfare reform, Universal Credit: a longitudinal controlled study. Lancet Public Health 2020;5:e157–64. 32113519 10.1016/S2468-2667(20)30026-8 PMC7208537

[32] Public Health England . Surveillance of influenza and other respiratory viruses in the United Kingdom: Winter 2013/14. London: Public Health England, 2015.

[33] Raleigh V . What is happening to life expectancy in the UK? London: The King’s Fund, 2019.

[34] Benach J , Vives A, Amable M et al. Precarious employment: understanding an emerging social determinant of health. Annu Rev Public Health 2014;35:229–53. 24641559 10.1146/annurev-publhealth-032013-182500

[35] Copeland A , Bambra C, Nylén L et al. All in it together? The effects of recession on population health and health inequalities in England and Sweden, 1991-2010. Int J Health Serv 2015;45:3–24. 26460444 10.2190/HS.45.1.b

[36] Curtis S et al. Changing labour market conditions during the ‘great recession’ and mental health in Scotland 2007–2011: an example using the Scottish longitudinal study and data for local areas in Scotland. Soc Sci Med 2019;227:1–9. 30219490 10.1016/j.socscimed.2018.08.003

[37] Minton J , Fletcher E, Ramsay J et al. How bad are life expectancy trends across the UK, and what would it take to get back to previous trends? J Epidemiol Community Health 2020;1–6. doi: 10.1136/jech-2020-213870. PMC7577090 32385127

[38] Barr B , Higgerson J, Whitehead M. Investigating the impact of the English health inequalities strategy: time trend analysis. BMJ 2017;358:1–8. 10.1136/bmj.j3310 PMC5527348 28747304

[39] Simpson L , Wilson T, Shalley F. The Shelf Life of Official Sub-National Population Forecasts in England. Appl Spat Anal Policy 2019. doi: 10.1007/s12061-019-09325-3.

[40] Shaw C . Fifty years of United Kingdom national population projections. Popul Trends 2007;128:8–23. 17691537

[41] Leon DA , Jdanov DA, Shkolnikov VM. Trends in life expectancy and age-specific mortality in England and Wales, 1970–2016, in comparison with a set of 22 high-income countries: an analysis of vital statistics data. Lancet Public Health 2019;4:e575–82. 31677776 10.1016/S2468-2667(19)30177-X

[42] Wearden G . Bank of England warns UK faces historic recession; US jobless claims hit 3.1m - business live. The Guardian, 2020. https://www.theguardian.com/business/live/2020/may/07/bank-of-england-interest-rates-covid-19-downturn-us-job-losses-business-live (1 September 2020, date last accessed).

