# Supplementary material for: Why were there 231 707 more deaths than expected in England between 2010 and 2018? An ecological analysis of mortality records
Source: J Public Health (Oxf). 2021 Mar 26;44(2):310–8. doi: 10.1093/pubmed/fdab023 (PMC8083632; doi:10.1093/pubmed/fdab023)
Supplement: Appendix_fdab023 [file appendix_fdab023.docx]

Appendix Explaining excess deaths at Local Authority level by sex, 2010-11 to 2017-18

|  | Model 1 | | | | Model 2 | | | | Model 3 | | | | Model 4 | | | | Model 5 | | | |
| --- | --- | --- | --- | --- | --- | --- | --- | --- | --- | --- | --- | --- | --- | --- | --- | --- | --- | --- | --- | --- |
|  | B | L CI | U CI | Sig | B | L CI | U CI | Sig | B | L CI | U CI | Sig | B | L CI | U CI | Sig | B | L CI | U CI | Sig |
| Intercept | | | | | | | | | | | | | | | | | | | | |
| (m) | 1.37 | -1.41 | 4.14 |  | -7.34 | -11.29 | -3.38 | *** | -9.85 | -13.88 | -5.82 | *** | -9.92 | -14.14 | -5.69 | *** | -9.38 | -13.46 | -5.30 | *** |
| (f) | 2.51 | -.0.23 | 5.26 | . | -5.26 | -9.29 | -1.23 | * | -6.60 | -10.74 | -2.45 | ** | -4.98 | -9.29 | -0.66 | * | -4.81 | -9.08 | -0.54 | * |
| Welfare reform: average estimated household loss | | | | | | | | | | | | | | | | | | | | |
| (m) | 0.00 | 0.00 | 0.01 | ** | 0.01 | 0.00 | 0.01 | *** | 0.01 | 0.00 | 0.01 | *** | 0.01 | 0.00 | 0.01 | *** | 0.02 | 0.01 | 0.03 | *** |
| (f) | 0.00 | 0.00 | 0.01 | *** | 0.01 | 0.00 | 0.01 | *** | 0.01 | 0.00 | 0.01 | *** | 0.01 | 0.00 | 0.01 | *** | 0.01 | 0.01 | 0.02 | *** |
| Proportion 65+ (2010) | | | | | | | | | | | | | | | | | | | | |
| (m) |  |  |  |  | 0.38 | 0.25 | 0.50 | *** | 0.47 | 0.34 | 0.60 | *** | 0.61 | 0.41 | 0.81 | *** | 0.52 | 0.33 | 0.72 | *** |
| (f) |  |  |  |  | 0.28 | 0.17 | 0.38 | *** | 0.30 | 0.19 | 0.41 | *** | 0.33 | 0.18 | 0.47 | *** | 0.29 | 0.15 | 0.43 | *** |
| Net change migration (16-29) | | | | | | | | | | | | | | | | | | | | |
| (m) |  |  |  |  |  |  |  |  | -45.94 | -91.65 | -0.23 | * | -42.08 | -88.12 | 3.96 | . | -34.73 | -79.26 | 9.80 |  |
| (f) |  |  |  |  |  |  |  |  | 13.83 | -25.72 | 53.38 |  | 4.48 | -36.47 | 45.42 |  | 7.59 | -32.94 | 48.11 |  |
| Net change migration (65+) | | | | | | | | | | | | | | | | | | | | |
| (m) |  |  |  |  |  |  |  |  | -323.16 | -477.88 | -168.43 | *** | -307.16 | -462.39 | -151.93 | *** | -203.78 | -359.13 | -48.43 | * |
| (f) |  |  |  |  |  |  |  |  | -187.11 | -334.22 | -39.99 | * | -188.47 | -335.06 | -41.87 | * | -133.20 | -282.83 | 16.43 | . |
| Change in neontic dependency ratio | | | | | | | | | | | | | | | | | | | | |
| (m) |  |  |  |  |  |  |  |  |  |  |  |  | -0.11 | -0.61 | 0.39 |  | -0.36 | -0.85 | 0.13 |  |
| (f) |  |  |  |  |  |  |  |  |  |  |  |  | -0.60 | -1.13 | -0.08 | * | -0.66 | -1.17 | -0.14 | * |
| Change in gerontic dependency ratio | | | | | | | | | | | | | | | | | | | | |
| (m) |  |  |  |  |  |  |  |  |  |  |  |  | -0.34 | -0.71 | 0.02 | . | -0.48 | -0.83 | -0.12 | ** |
| (f) |  |  |  |  |  |  |  |  |  |  |  |  | -0.18 | -0.50 | 0.15 |  | -0.27 | -0.59 | 0.06 |  |
| IMD (2010) average rank | | | | | | | | | | | | | | | | | | | | |
| (m) |  |  |  |  |  |  |  |  |  |  |  |  |  |  |  |  | 0.00 | 0.00 | 0.00 | *** |
| (f) |  |  |  |  |  |  |  |  |  |  |  |  |  |  |  |  | 0.00 | 0.00 | 0.00 | ** |
|  | R^2^ | | Adjusted R^2^ | | R^2^ | | Adjusted R^2^ | | R^2^ | | Adjusted R^2^ | | R^2^ | | Adjusted R^2^ | | R^2^ | | Adjusted R^2^ | |
| (m) | 2.87 | | 2.57 | | 12.14 | | 11.59 | | 18.06 | | 17.04 | | 19.01 | | 17.48 | | 24.8 | | 23.14 | |
| (f) | 2.99 | | 2.69 | | 10.07 | | 9.51 | | 11.89 | | 10.79 | | 13.68 | | 12.05 | | 15.93 | | 14.07 | |
| Signif. codes: 0 ‘***’ 0.001 ‘**’ 0.01 ‘*’ 0.05 ‘.’ 0.1 ‘ ’ 1; Note: *n* = 325 Local Authorities, Isles of Scilly excluded due to missing data | | | | | | | | | | | | | | | | | | | | |
